# Vitamin D_3_ preserves blood retinal barrier integrity in an *in vitro* model of diabetic retinopathy

**DOI:** 10.3389/fphar.2022.971164

**Published:** 2022-08-26

**Authors:** Francesca Lazzara, Anna Maria Longo, Giovanni Giurdanella, Gabriella Lupo, Chiara Bianca Maria Platania, Settimio Rossi, Filippo Drago, Carmelina Daniela Anfuso, Claudio Bucolo

**Affiliations:** ^1^ Department of Biomedical and Biotechnological Sciences, School of Medicine, University of Catania, Catania, Italy; ^2^ Faculty of Medicine and Surgery, University of Enna “Kore”, Enna, Italy; ^3^ Center for Research in Ocular Pharmacology–CERFO, University of Catania, Catania, Italy; ^4^ Multidisciplinary Department of Medical, Surgical and Dental Sciences, University of Campania “Luigi Vanvitelli”, Naples, Italy

**Keywords:** vitamin D_3_, blood retinal barrier, diabetic retinopathy, inflammation, angiogenesis, P2X7R

## Abstract

The impairment of the blood retinal barrier (BRB) represents one of the main features of diabetic retinopathy, a secondary microvascular complication of diabetes. Hyperglycemia is a triggering factor of vascular cells damage in diabetic retinopathy. The aim of this study was to assess the effects of vitamin D_3_ on BRB protection, and to investigate its regulatory role on inflammatory pathways. We challenged human retinal endothelial cells with high glucose (HG) levels. We found that vitamin D_3_ attenuates cell damage elicited by HG, maintaining cell viability and reducing the expression of inflammatory cytokines such as IL-1β and ICAM-1. Furthermore, we showed that vitamin D_3_ preserved the BRB integrity as demonstrated by trans-endothelial electrical resistance, permeability assay, and cell junction morphology and quantification (ZO-1 and VE-cadherin). In conclusion this *in vitro* study provided new insights on the retinal protective role of vitamin D_3_, particularly as regard as the early phase of diabetic retinopathy, characterized by BRB breakdown and inflammation.

## Introduction

The etiopathogenesis of diabetic retinopathy is still not fully elucidated and several pathways are involved in the exacerbation of this pathological condition. Oxidative stress, inflammation, and vascular dysfunction affect the integrity of inner blood retinal barrier (iBRB composed, among others, by pericytes, endothelial cells and Müller cells) and the outer blood retinal barrier (oBRB composed by retinal pigment epithelium RPE). Moreover, the upregulation of some proangiogenic factors such as vascular endothelial growth factor-A (VEGF-A), leads to retinal ischemia and blood retinal barrier (BRB) impairment (Bucolo and Drago, 2004; Tarr et al., 2013; Duh et al., 2017; Lazzara, 2022; Shukla and Tripathy, 2022). The iBRB and oBRB modulate the transport of molecules regulating the permeability across the retinal endothelium and the pigmented epithelial cells, respectively. Tight junctions (TJs) and adherens junctions are multiple junctional protein complexes endowed of regulation of BRB integrity, which is strongly altered by high plasmatic levels of glucose. Hyperglycemia causes retinal micro-vasculopathy, inflammation, and retinal neurodegeneration (Gui et al., 2020). The activation of toll-like receptors 4 (TLR-4), which leads to the over expression of inflammatory markers, such as IL-1β (Cao et al., 2021; Bayan et al., 2022), is one of the diabetes-associated retinal alterations (Wang et al., 2015). It has been demonstrated that the upregulation of IL-1β in retinal endothelial cells is induced by hyperglycemia (Demircan et al., 2006; Liu et al., 2012; Wooff et al., 2019). Moreover, IL-1β is also a stronger inducer of other inflammatory cytokines through the activation of p38MAPK/NF-κB pathway (Liu et al., 2015). High glucose levels represent a strong stimulus that triggers the phosphorylation/activation of ERK proteins, in retinal endothelial cells (Liu et al., 2014; Liu et al., 2015; Lazzara et al., 2019). All these diabetic-related events are correlated to the up-regulation of ICAM-1, induced by both angiogenic (overexpression of VEGF-A) and inflammatory stimuli (up-regulation of inflammatory cytokines). In fact, retinal endothelial cells are the main producers of ICAM-1, which exacerbates the microvascular leukostasis, i.e., the adhesion and transmigration of leukocytes to endothelium, in diabetic retinopathy (DR) (Joussen et al., 2003; van der Wijk et al., 2017). Vitamin D_3_ is a fat-soluble steroid hormone, endogenously produced by the human body, acting as a nuclear hormone, it has the highest affinity for the vitamin D receptor (VDR). VDR is ubiquitously expressed in the cells of the whole human body, and it is expressed in retinal cells, including endothelial cells. Vitamin D_3_ has genomic (through vitamin D response element, VDRE, on target genes) and nongenomic effects and these last are related to its activity on protein kinases, including MAPKs (Revelli et al., 1998; Hii and Ferrante, 2016; Jamali et al., 2018). Interestingly, it has been demonstrated that VDR is expressed both in vascular endothelial cells and pericytes, and the effects of vitamin D_3_ on vascular cells is still object of several studies (Jamali et al., 2018; Jamali et al., 2019).

In the present study we investigated the effects of vitamin D_3_ on primary retinal endothelial cells, challenged with high glucose levels. We demonstrated the anti-inflammatory and anti-angiogenic activity of this vitamin in an *in vitro* model of DR, showing its efficacy at reducing DR-related BRB loss of integrity. Our results suggest new insight for potential therapeutic implications of vitamin D_3_ for the management of early stages DR.

## Methods

### Cell culture

Human retinal endothelial cells (HRECs) were purchased from Innoprot^®^ (Derio – Bizkaia, Spain). Cells were cultured at 37°C, in humidified atmosphere (5% CO_2_), in Endothelial Cell Medium (ECM) supplemented with 5% fetal bovine serum (FBS), 1% ECGS (Endothelial Cell Growth Supplement) and 100 U/ml penicillin 100 μg/ml streptomycin, in flask precoated with fibronectin (1 mg/ml) (Innoprot, Derio – Bizkaia, Spain) for 1 h at 37°C. After reaching confluence (approximately 70%), cells were used for experimental procedures. All the treatments were carried out in medium containing 2.5% FBS. Cells growth in medium containing 5 mM glucose (physiological glucose concentration) served as control group. HRECs were also exposed to medium containing 40 mM glucose (high glucose, HG) (Huang et al., 2016; Lazzara et al., 2019) obtained from the basal glucose concentration of medium (5 mM) with the addition of 35 mM of glucose on basis of used final volume. HRECs were pre-treated for 24 h with vitamin D_3_ (1 µM) and then were exposed to HG with or without vitamin D_3_ for 24, 48 and 72 h.

### MTT

The 3-[4,5-dimethylthiazol-2-yl]-2,5-diphenyl tetrasodium bromide (MTT; Chemicon, Temecula, CA, United States) was used to assess cell viability after HG (40 mM) challenge and vitamin D_3_ (1 µM) treatment. Optimal cell density was obtained by seeding 1,5 × 10^4^ cells/well in 96-well plates (Costar, Corning, NY, United States). After pretreatment with vitamin D_3_ HRECs were subjected to co-treatment in a fresh medium for 24 and 48 h with vitamin D_3_ (1 μM) and HG (40 mM). At the end of the treatment, HRECs were incubated at 37°C with MTT (0.5 mg/ml) for 3 h; then DMSO was added, and absorbance was measured at 570 nm in a plate reader (VariosKan, Thermo Fisher Scientific, Waltham, MA, United States). Graphs were built converting absorbance (abs) to viability (% of control) using the following equation (abs_x_ ÷ abs_ctrl−_) × 100, where abs_x_ is absorbance in the x well, and abs_ctrl−_ is the average absorbance of negative control cells (untreated cells).

### Lactate dehydrogenase

Lactate dehydrogenase (LDH) cell release was measured using the Cytotoxicity Detection KitPLUS (LDH) (ROCHE, Mannheim, Germany). HRECs cells were seeded at 1,5 × 10^4^ cells/well in 96-well plates (Costar, Corning, NY, United States). After pretreatment with vitamin D_3_, HRECs were subjected to co-treatment in a fresh medium for 24 and 48 h with vitamin D_3_ (1 μM) and HG (40 mM). After these time points, according to manufacturer’s protocol, lysis solution was added to positive control wells (non-treated cells) for 15 min. After transferring 100 μl of medium in a new multi-well plate, 100 μl of working solution was added. After 10–15 min at room temperature, at last, 50 μl of stop solution was added. The absorbance values were measured at 490 nm using a plate reader (VarioSkan, Thermo Fisher Scientific, Waltham, MA, United States). LDH release is reported as LDH (% control) (abs_x_ ÷ abs_ctrl+_) × 100. In the equation, abs_x_ is absorbance in the x well and abs_ctrl+_ is the average absorbance of positive control cells (untreated lysed cells). Absorbance values were corrected by subtracting medium absorbance.

### Blood retinal barrier integrity assessment

The effect vitamin D_3_ and HG challenge on BRB integrity was evaluated by measurements of TEER, by using a Millicel-Electrical Resistance System (ERS2) (Merck, Millipore, Burlington, MA, United States ) as previously described (Giurdanella et al., 2017; Fresta et al., 2020). To evaluate the modification of paracellular permeability under the above-mentioned conditions, the luminal-to-abluminal movements of Na-F, across endothelial cell monolayers, were measured by using a Varioskan Flash microplate reader (Thermo Fisher Scientific, Waltham, MA, United States ) as previously described (Fresta et al., 2020).

### Immunocytochemistry

ZO-1 immunodetection was carried out as follows. Glass chamber slides were coated with a fibronectin for 1 h at 37°C and washed with sterile water. HRECs (6 × 10^4^ cells/well) were seeded on 24-well fibronectin coated glass chamber slides. Cells were incubated for 4 days at 37°C in a 5% CO_2_ humidified atmosphere. Cell adhesion and confluence was reached within 5 days and the medium was changed every 2 days. Cells were shifted for 24 h with vitamin D_3_ and for 48 h to a medium containing 40 mM glucose (HG), with or without vitamin D_3_. HRECs growth in medium with physiological glucose concentration (5 mM) served as control. After 48 h, cells were fixed with ice-cold acetone for 15 min and with ice-cold methanol for 20 min. Thereafter, cells were washed with cold phosphate buffered saline (PBS, pH 7.4) and blocked with 5% normal goat serum (NGS) and 0.1% Triton X-100 in PBS solution, for 30 min at room temperature. Cells were then incubated overnight at 4°C with primary antibody against ZO-1 (dilution 1:100, rabbit monoclonal; catalog n. 61-7300, Life Technology, Monza, Italy). After overnight incubation and primary antibody washout with PBS, the secondary anti-rabbit Alexa 488-coniugated antibody (dilution 1:200, Life Technology, Monza, Italy) was added for 1 h at room temperature in the dark. VE-cadherin immunodetection was carried out with a different protocol. HRECs (6 × 10^4^ cells/well) were seeded on 24-well fibronectin coated glass chamber slides pre-coated with fibronectin for 1 h at 37°C and then incubated for 4 days at 37°C in a 5% CO_2_ humidified atmosphere. The medium was changed every 2 days. Thereafter, the cells were shifted to different medium, as described for ZO-1 staining. After 48 h of treatment the cells were fixed with 4% paraformaldehyde for 15 min at room temperature, washed twice with cold PBS and permeabilized with 0.3% Triton X-100 in PBS (pH 7.4) for 5 min at room temperature. After blocking with 1% bovine serum albumin (BSA) in PBS for 1 h, the cells were incubated with the rabbit anti-VE-cadherin antibody (1:100, Catalog n. 2500 Cell signaling, Technology, Danvers, MA, United States ) in 1% BSA-PBS solution, overnight at 4°C. Then, the slides were washed three times with PBS and 1 h incubation was carried out with anti-rabbit Alexa 488-coniugated secondary antibody (1:200 dilution, Life Technologies, Monza, Italy), at room temperature in the dark. For p-NFκB p65 immunostaining HRECs were plated at a density of 4 × 10^4^ in 24-well glass chamber slides pre-coated with fibronectin for 1 h at 37°C and then incubated for 3 days at 37°C in a 5% CO_2_ humidified atmosphere. Thereafter, the cells were pretreated for 24 h with vitamin D_3_ and for 24 h with high glucose. Then, the cells were fixed with 4% paraformaldehyde for 15 min at room temperature, washed twice with cold PBS and permeabilized with 0.2% Triton X-100 in PBS (pH 7.4) for 15 min at room temperature. After blocking with 5% NGS and 0.3% Triton X-100 in PBS solution, for 30 min at room temperature, the cells were incubated with the mouse-anti- phospho-NFκB p65 (Ser536; 1:200, Catalog n. 3036 Cell signaling, Technology, Danvers, MA, United States ) in 1% NGS and 0.2% Triton X-100 in PBS solution overnight at 4°C. After overnight incubation, the slides were washed three times with PBS. Then, 1 h incubation was carried out with anti-mouse IgG H + L (Dylight 550) secondary antibody in 0.1% Triton X-100 in PBS (1:300 dilution, Abcam, Cambridge, United Kingdom), at room temperature in the dark. Nuclei staining was carried out for 10 min with 4′,6-diamidino-2-phenylindole (DAPI) (1:10.000; D1306, Life Technologies, Monza, Italy). Finally, the slides were mounted using mounting medium (Life Technologies, Monza, Italy). Images were acquired with a fluorescence microscope Zeiss Observer Z1 equipped with the Apotome.2 acquisition system connected to a digital camera (Carl Zeiss, Oberkochen, Germany). Images were acquired at 40×. Semi-quantitative evaluation of junction protein expression was carried out analyzing images from slides of each condition *n* = 4 (5 mM glucose, 40 mM glucose, 40 mM glucose + 1 µM vitamin D_3_). The images (*n* = 4 per group) were analyzed by two investigators unaware of experimental design.

### Extraction of total ribonucleic acid and cDNA synthesis

Extraction of total RNA, from HREC cells was performed, after 72 h of treatment, with a TRIzol Reagent (Invitrogen, Life Technologies, Carlsbad, CA, United States). The A_260_/A_280_ ratio of optical density of RNA samples (measured with Multimode Reader Flash di Varioskan™) was 1.95–2.01; this RNA purity was confirmed with the electrophoresis in non-denaturing 1% agarose gel (in TAE). cDNA was synthesized from 2 μg RNA with a reverse transcription kit (SuperScript™ II Reverse transcriptase, Invitrogen, Thermo Fisher Scientific, Carlsbad, CA, United States).

### qRT-PCR

Real-time PCR was carried out with the Rotor-Gene Q (Qiagen). The amplification reaction mix included the Master Mix Qiagen (10 μl) (Qiagen QuantiNova SYBR Green Real-Time PCR Kit) and cDNA (1 μl, 100 ng). Forty-five amplification cycles were carried out for each sample. Results were analyzed with the 2^−ΔΔCt^ method. Quantitative PCR experiments followed the MIQE guidelines (Bustin et al., 2009). Gene expression levels were normalized with levels of housekeeping gene (18S). Primers were purchased from Eurofins Genomics (Milan, Italy). Forward and reverse primer sequences are herein listed: IL-1β (forward: 5′-AGC​TAC​GAA​TCT​CCG​ACC​AC-3′; reverse: 5′-CGT​TAT​CCC​ATG​TGT​CGA​AGA​A-3′), VEGF-A (forward 5′-AGG​GCA​GAA​TCA​TCA​CGA​AG-3’; reverse 5′-ATC​CGC​ATA​ATC​TGC​ATG​GT-3′), 18S (forward 5′-AGT​CCC​TGC​CCT​TTG-3’; reverse 5′-GAT​CCG​AGG​GCC​TCA​CTA​AAC-3′), ICAM-1 (forward 5′-ATG​CCC​AGA​CAT​CTG​TGT​CC-3′; reverse 5′-GGG​GTC​TCT​ATG​CCC​AAC​AA-3′), TLR-4 forward 5′-ATA​TTG​ACA​GGA​AAC​CCC​ATC​CA-3′; reverse 5′-AGA​GAG​ATT​GAG​TAG​GGG​CAT​TT-3′.

### Western blot

HRECs were cultured in 60 mm Petri dishes (4 × 10^5^). Proteins of whole cell lysates were extracted with RIPA Buffer, including protease and phosphatase inhibitors cocktail (Sigma-Aldrich, St. Louis, MO, United States). Total protein content, in each cell lysate sample, was determined by means of the BCA Assay Kit (Pierce™ BCA Protein Assay Kit, Invitrogen, Life Technologies, Carlsbad, United States). Extracted proteins (30 μg) were loaded on 4%–12% tris–glycine gel. After electrophoresis, proteins were transferred into a nitrocellulose membrane (Invitrogen, Life Technologies, Carlsbad, CA, United States). Membranes were blocked with milk, 5% Trisbuffered saline, and 0.2% Tween 20 (TBST) for 1 h at room temperature. Membranes were incubated overnight (4°C) with appropriate primary phospho-p44/42 MAPK (Rabbit, phospho-Erk1/2, 1:500 dilution, Catalog n. 9101 Cell Signaling Technology, Danvers, MA, United States ), p44/42 MAPK (Rabbit, Erk1/2, 1:500 dilution, Catalog n. 9102 Cell Signaling Technology, Danvers, MA, United States ) and anti-GAPDH (Rabbit mAb, 1:500 dilution, Catalog n. 2118 Cell Signaling Technology, Danvers, MA, United States ) antibodies. After overnight incubation, the membranes were then incubated with secondary chemiluminescent antibody (ECL anti-rabbit, 1:2000 dilution, NA934) for 1 h at room temperature. After secondary antibody, the membranes were incubated with ECL (SuperSignal™ West Pico PLUS Chemiluminescent Substrate, Thermo Fisher Scientific, Carlsbad, CA, United States) and were detected through I-Bright™ 1500 (Invitrogen, Life Technologies, Carlsbad, CA, United States) by using chemiluminescence. Densitometry analyses of blots were performed at non-saturating exposures and analyzed using ImageJ software (NIH, Bethesda, MD). Values were normalized to GAPDH, which was also used as loading control.

### *In vitro* tube formation assay

Tube formation assay was performed *in vitro* with Matrigel Basement Membrane Matrix system (BD, Bedford). The experimental protocol was run according to the manufacturer’s instructions. Gel solution was thawed at 4°C overnight, then 96-well plates were coated with 50 µl of Matrigel/well and allowed to solidify at 37°C for 2 h. HRECs were seeded at 15,000 cells per well in 50 µl assay medium, with or without HG and/or 1 µM vitamin D_3_. Each condition was run in triplicate. After 8 h of incubation, tube-like structures were photographed by using an inverted microscope. The total tube length was quantified with the ImageJ software (NIH, Bethesda, MD).

### Statistical analysis

Statistical analysis and graphs design were carried out with GraphPad Prism (GraphPad Software, La Jolla, CA, United States ). Data are reported as mean ± SD. One-way ANOVA, followed by Tukey-Kramer post-hoc test, was carried out for multiple comparisons. Post-hoc test was carried out given an F with *p* < 0.05, and no significant variance inhomogeneity was found within groups. Differences between groups were considered significant at *p* < 0.05.

## Results

### Cell viability and lactate dehydrogenase release

**FIGURE 1:**
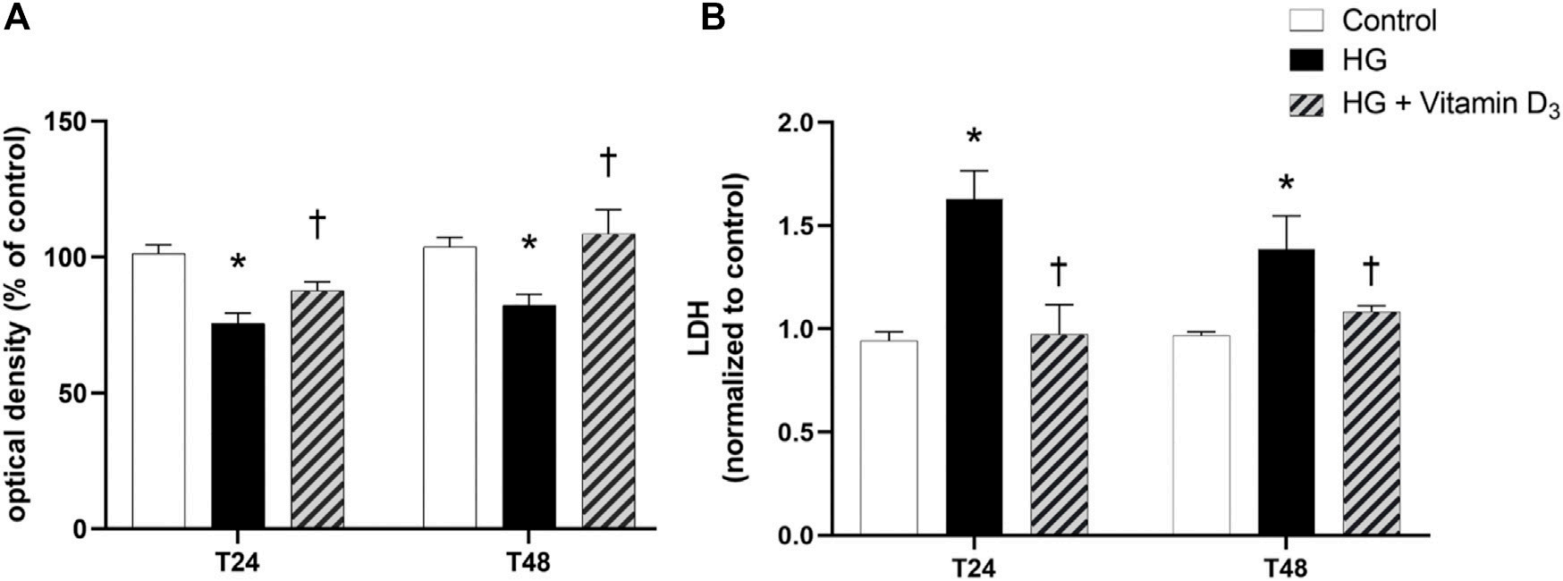
Vitamin D_3_ shows protective effect in HREC cells against high glucose (HG)-induced damage. Cells were pretreated for 24 h with vitamin D_3_ (1 µM) and for 24 and 48 h with HG (40 mM). At the end of treatment MTT **(A)** and LDH **(B)** assay were carried out. Values are reported as mean ± SD (*n* = 4). Data were analyzed by one-way ANOVA and Tukey’s post hoc test for multiple comparisons. *p < 0.05 vs. control; †*p* < 0.05 vs. HG.

After 24 and 48 h, high glucose induced a significant (*p* < 0.05) cell toxicity in terms of reduction of cell viability, in comparison to control (roughly 26% and 21% after 24 and 48 h, respectively) (Figure 1A). Pre-treatment with vitamin D_3_ (1 µM) significantly (*p* < 0.05) attenuates cell toxicity after 24 and 48 h, compared to high glucose treated cells (roughly 16% and 32% after 24 and 48 h, respectively). The same profile was observed in terms of LDH release (Figure 1B).

### Inner blood retinal barrier integrity

**FIGURE 2:**
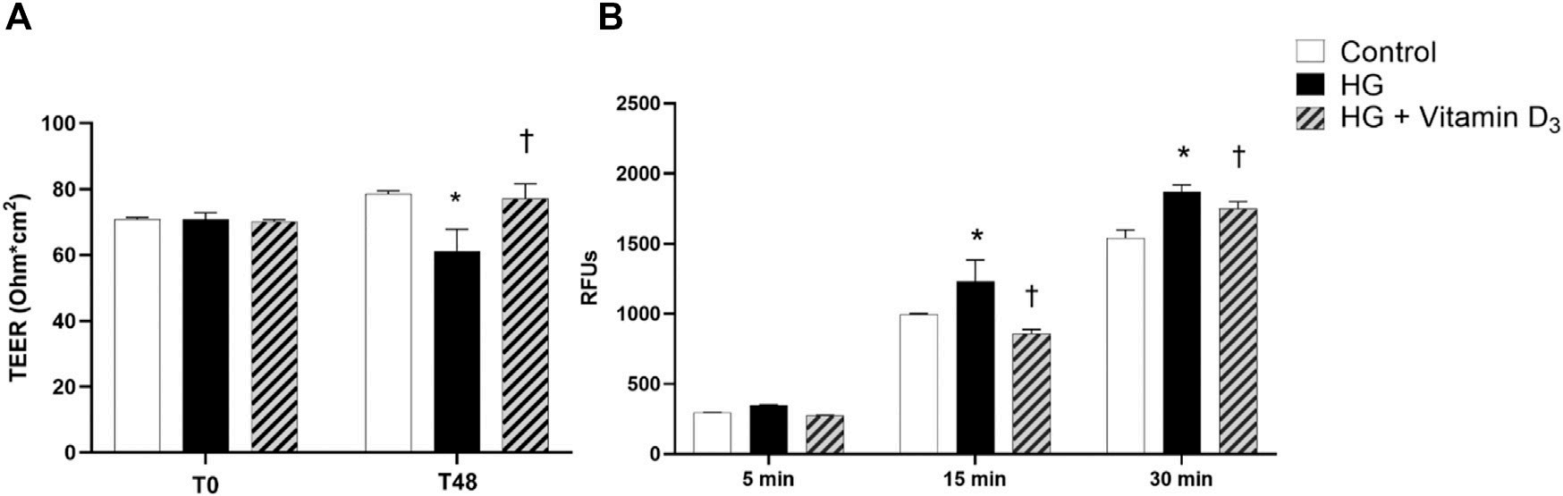
Vitamin D_3_ protects HREC cell monolayer from HG (high glucose)-induced damage. HRECs were pretreated with vitamin D_3_ (1 μM) for 24 h and co-treated with HG (40 mM) for 48 h. **(A)** Vitamin D_3_ increased TEER values, which were reduced by HG challenge after 48 h. **(B)** Measurement of apical-to basolateral Na-F permeability after 5, 15 and 30 min. Values are reported as mean ± SD; *n* = 4. Data were analyzed by one-way ANOVA and Tukey post-hoc test for multiple comparisons. *p < 0.05 vs. control; †*p* < 0.05 vs. HG.

To evaluate vitamin D_3_ effects on iBRB integrity, we measured trans endothelial electric resistance (TEER), a parameter of barrier permeability in cell cultures. We found TEER values significantly (*p* < 0.05) reduced (22%) after 48 h of high glucose damage, compared to control cells (Figure 2A). On the contrary, vitamin D_3_ treated cells showed significant (*p* < 0.05) increased TEER values, superimposable with control group (Figure 2A). These data were supported by the measurement of apical-to-basolateral permeability of sodium fluorescein (Na-F), a spectrophotometric approach for the assessment of cell monolayer permeability. Treatment with vitamin D_3_ (1 μM) was able to significantly (*p* < 0.05) preserve monolayer permeability (15’ and 30’) elicited by HG (Figure 2B).

**FIGURE 3:**
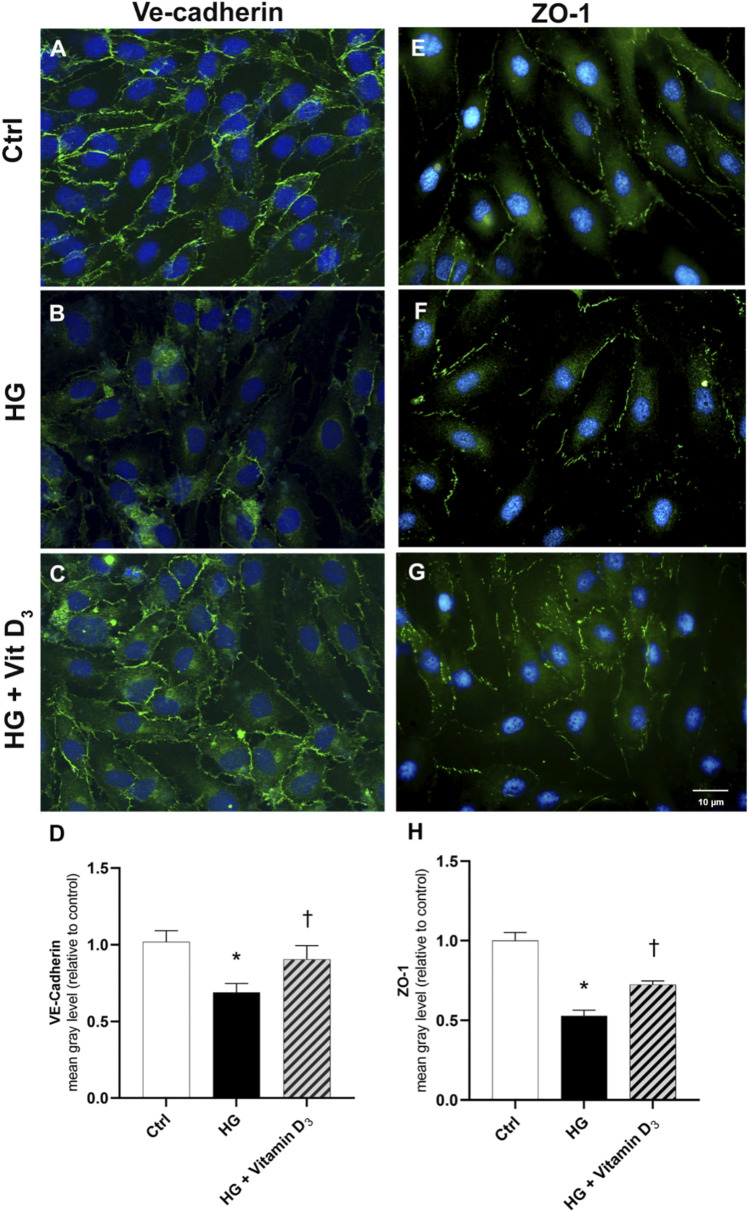
Vitamin D_3_ re-establishes iBRB integrity through modulation of VE-cadherin and ZO-1. HRECs were pretreated with vitamin D_3_ (1 μM) for 24 h and subsequently co-treated with HG (40 mM) for other 48 h. Vitamin D_3_ increased the expression of VE-cadherin and ZO-1 proteins, which were significantly reduced by HG. Representative images for VE-cadherin **(A,B,C)** and ZO-1 **(E,F,G)** expression in HRECs after treatment with HG and vitamin D_3_. VE-cadherin and ZO-1 were labeled with FITC (green); nuclei were labeled with DAPI (blue). Images were acquired at × 40 magnification. Scale bar: 10 µm. Fluorescence semi-quantification of VE-cadherin **(D)** and ZO-1 **(H)** protein (mean grey levels). Values are reported as mean ± SD; *n* = 4. Data were analyzed by one-way ANOVA and Tukey post-hoc test for multiple comparisons. *p < 0.05 vs. control; †*p* < 0.05 vs. HG.

Since BRB integrity is related to the expression and cell membrane localization of tight junction (TJ) proteins such as ZO-1 and adherens junction such as VE-cadherin (AJ), the expression of this proteins was analyzed by immunocytochemistry (Figure 3). High glucose damage significantly (*p* < 0.05) decreased the expression of both proteins in HRECs, compared to control cells (roughly 33% and 47% of VE-cadherin and ZO-1, respectively) (Figure 3). On the other hand, pre-treatment with vitamin D_3_ protected HRECs from HG-damage preserving the expression of ZO-1 and VE-cadherin after 48 h of exposure to HG and vitamin D_3_ (roughly 32% and 37% of VE-cadherin and ZO-1, respectively) (Figure 3).

**FIGURE 4:**
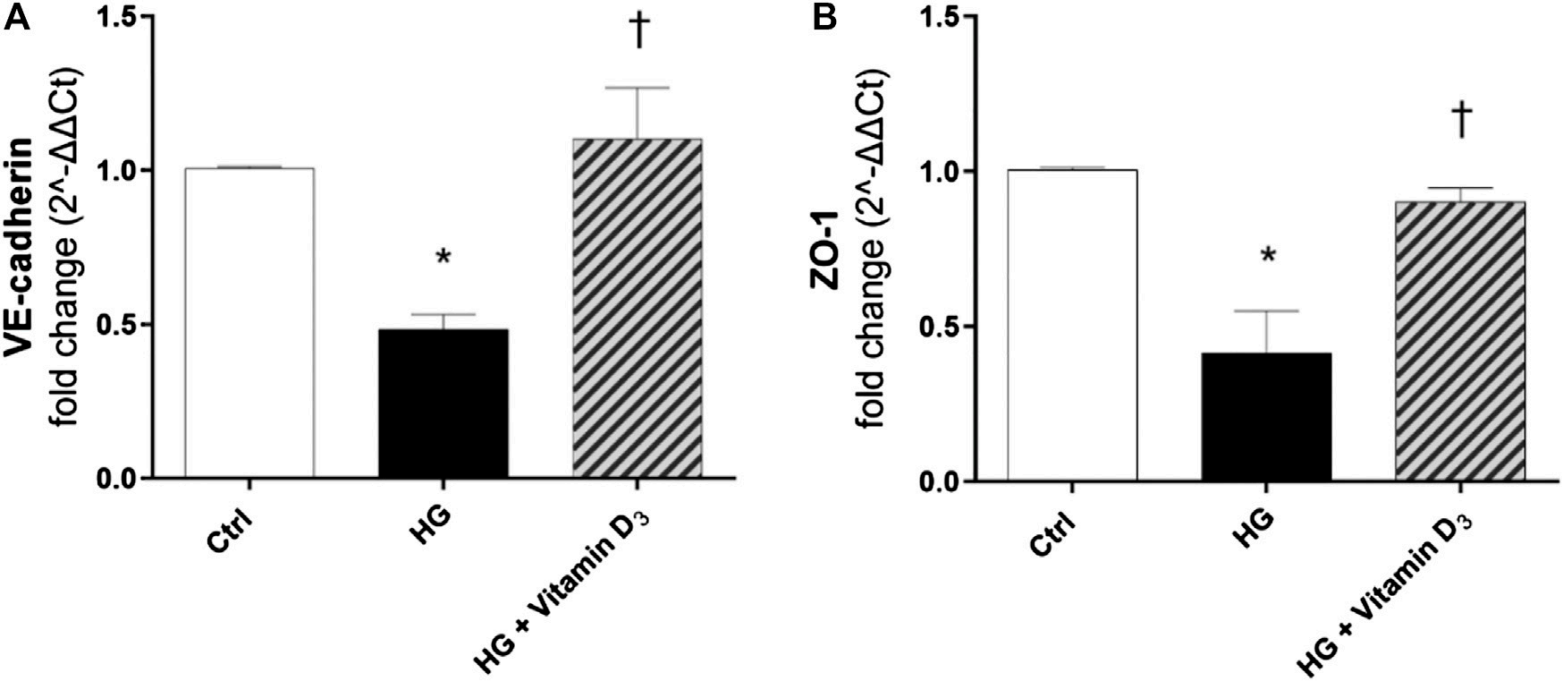
Vitamin D_3_ induces VE-cadherin and ZO-1 mRNA expression. VE-cadherin **(A)** and ZO-1 **(B)** mRNA levels after treatment with vitamin D_3_ and HG for 48 h. Values are reported as mean ± SD; *n* = 4. Data were analyzed by one-way ANOVA and Tukey post-hoc test for multiple comparisons. *p < 0.05 vs. control; †*p* < 0.05 vs. HG.

Further, we analyzed at transcriptional level the modulation of VE-cadherin and ZO-1. After 48 h of HG exposure, ZO-1 and VE-cadherin mRNAs levels were significantly (*p* < 0.05) down-regulated in HRECs (roughly 0.6-fold and 0.5-fold for VE-cadherin and ZO-1, respectively), while pre-treatment with vitamin D_3_ reverted this effect (Figures 4A,B), maintaining levels of mRNA expression to control values.

### Inflammatory process modulation

**FIGURE 5:**
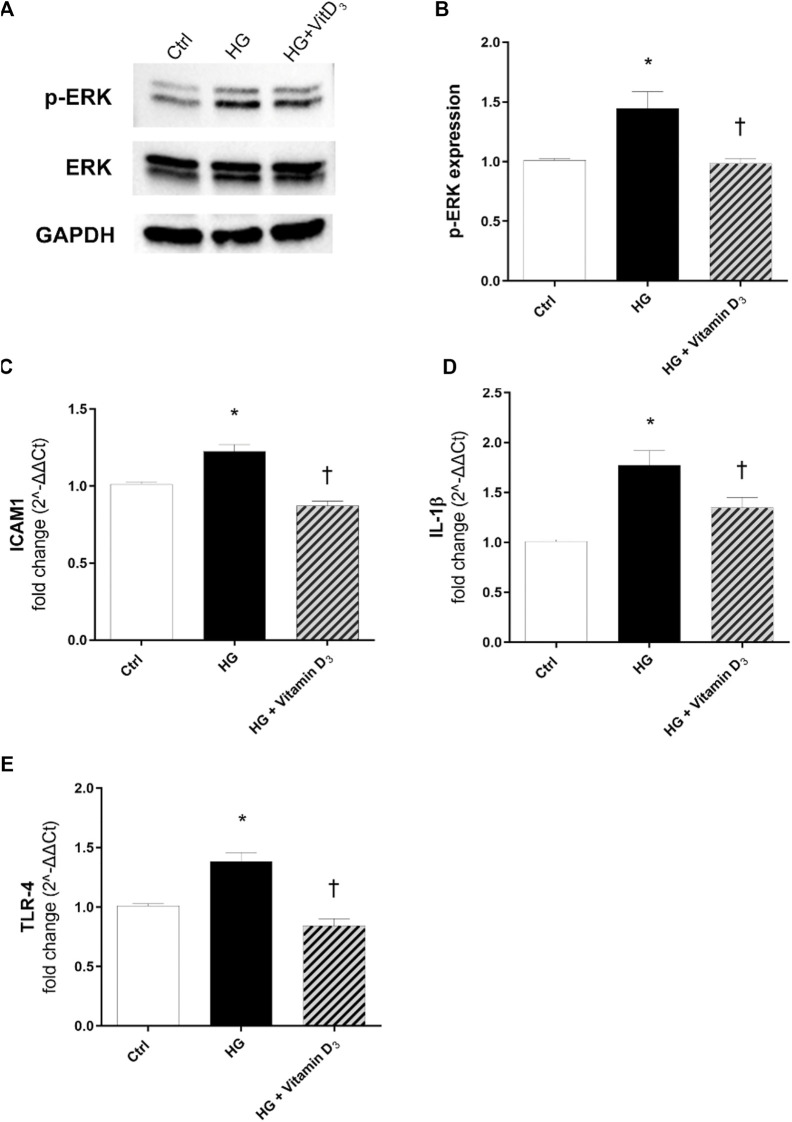
Vitamin D_3_ counteracts inflammation and angiogenesis in HREC after HG-induced damage. Vitamin D_3_ effect on the inflammatory pathway activated by high glucose (HG) in HRECs. **(A)** Immunoblot analysis of ERK1/2 phosphorylation in lysates from HRECs, pre-treated for 24 h with vitamin D_3_ (1 µM) and subsequently co-treated with HG (40 mM) for other 24 h. **(B)** Bar graphs show the densitometry analysis of each band, carried out with the Image J program, p-ERK densitometry has been normalized to total ERK values. The effect of HG and vitamin D_3_ at mRNA levels was evaluated after 72 h of HG challenge. The treatment with vitamin D_3_ reduced ICAM-1 **(C)**, IL-1β **(D)**, TLR4 **(E)** mRNA expression. The mRNA levels were evaluated by qPCR. Each bar represents the means ± SD (*n* = 4; each run in triplicate). *p < 0.05 vs. control; †*p* < 0.05 vs. HG.

After 24 h, HG exposure elicited a significant (*p* < 0.05) increase of phosphorylated ERK protein, in comparison to control cells (Figures 5A,B). As shown in Figures 5 (A,B), vitamin D_3_ (*p* < 0.05) led to a significant (*p* < 0.05) reduction of ERK phosphorylation (0.5-fold of reduction compared to HG). Furthermore, we analyzed mRNA expression of inflammatory cytokines after 24 and 48 h of HG challenge, but although the trend was rising, data were not significant (data not shown). Instead, we found that after 72 h, HG challenge induced a significant (*p* < 0.05) up-regulation of ICAM-1 and IL-1β (Figures 5C,D), whose mRNA levels were significantly (*p* < 0.05) reduced by vitamin D_3_ treatment (0.4-fold compared to HG-treated cells). Further, TLR-4 mRNA levels were higher in HRECs challenged with HG, compared to control cells (Figure 5E), and vitamin D_3_ treatment restored TLR-4 mRNA to control cell levels (0.5-fold compared to HG) (Figure 5E).

**FIGURE 6:**
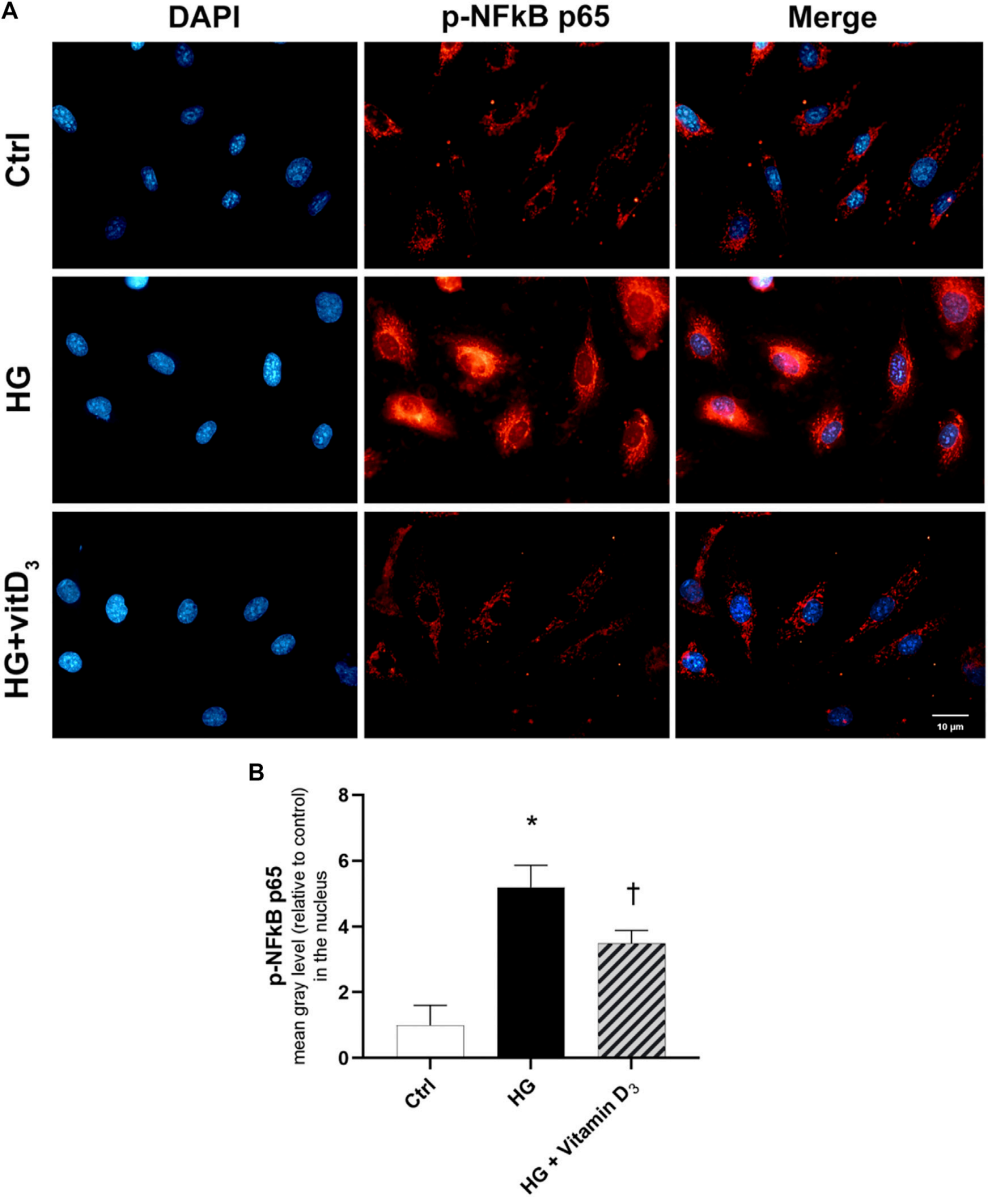
Effect of vitamin D_3_ on NF-κB activation in HG-challenged HRECs. **(A)** Representative images of phosphorylated p-NFκB p65 (red) translocation into the nuclei (blue) stained with DAPI. HRECs were pre-treated with vitamin D_3_ (1 µM) for 24 h and then with or without high glucose for 24 h. **(B)** Fluorescence semi-quantification of p-NFκB p65 protein (mean grey levels) into the nucleus. Nuclei were labeled with DAPI (blue). Images were acquired at × 40 magnification. Scale bar: 10 µm. Values are reported as mean ± SD; *n* = 4. Data were analyzed by one-way ANOVA and Tukey post-hoc test for multiple comparisons. *p < 0.05 vs. control; †*p* < 0.05 vs. HG.

Finally, we evaluated the effects of vitamin D_3_ on NFκB activation and nuclear translocation, to confirm the anti-inflammatory activity of vitamin D_3_ in HRECs after 24 h of HG exposure. HG induced the nuclear translocation of the phosphorylated p65 subunit of NFκB, as shown in Figure 6A. NFκB activation and translocation was significantly (*p* < 0.05) counteracted by the pre-treatment with vitamin D_3_, inhibiting the p65 nuclear translocation (Figures 6A,B).

### Anti-angiogenic activity

**FIGURE 7:**
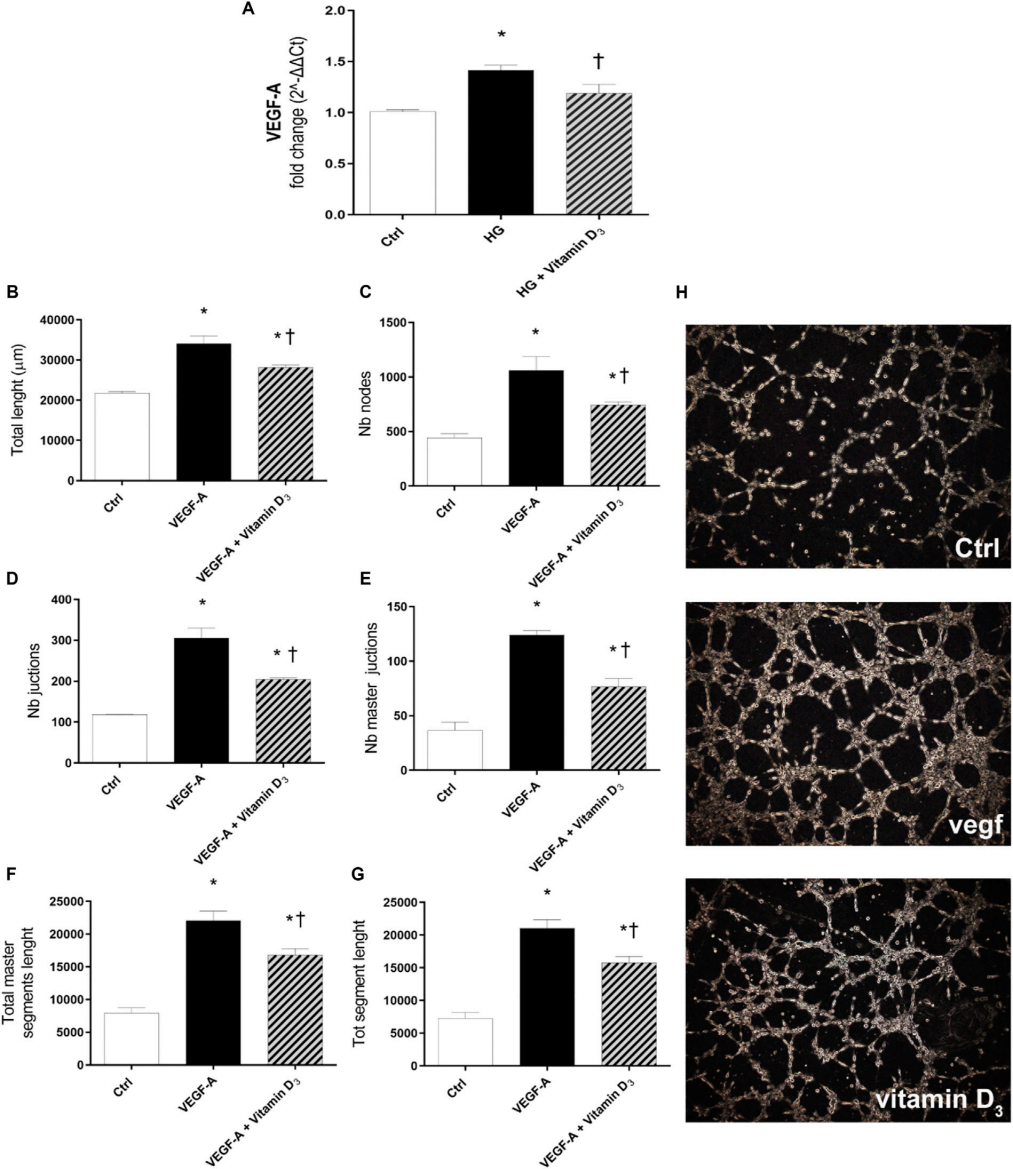
Effect of vitamin D_3_ on angiogenesis. **(A)** Real-time PCR; VEGF-A mRNA expression. HRECs were pre-treated with vitamin D_3_ (1 µM) for 24 h and then with or without HG (40 mM) for 72 h. Vitamin D_3_ decreased mRNA levels of VEGF-A and exerted antiangiogenic activity. **(B–G)** Quantification of total tube length, nb nodes, nb junctions, Nb master junctions, total master segments length and total segment length, was carried out using the Angiogenesis Analyzer tool for ImageJ software. HRECs were treated with 80 ng/ml VEGF-A in presence or absence of vitamin D_3_ (1 µM). **(H)** Representative optical phase-contrast micrographs of tubelike structures (× 40 magnification) observed in the tube formation assays (Matrigel) after 8 h. Values are reported as mean ± SD; *n* = 4. Data were analyzed by one-way ANOVA and Tukey post-hoc test for multiple comparisons. *p < 0.05 vs. control; †*p* < 0.05 vs. HG or VEGF-A.

After 72 h of HG challenge, retinal endothelial cells expressed significant (*p* < 0.05) higher levels of VEGF-A, compared to control cells (Figure 7A). The treatment with vitamin D_3_ significantly (*p* < 0.05) reduced VEGF-A mRNA levels, in comparison to cells exposed to HG (Figure 7A). Furthermore, to confirm the anti-angiogenic effect of vitamin D_3_, we carried out the tube-formation Matrigel assay (Figures 7B–H), as previously used for the evaluation of angiogenic potential of HRECs (Yadav et al., 2012; Giurdanella et al., 2017; Platania et al., 2020). Vitamin D_3_ exerted a significant (*p* < 0.05) anti-angiogenic activity on HRECs treated with 80 ng/ml VEGF-A (Figures 7B–H). In particular, vitamin D_3_ significantly (*p* < 0.05) decreased the number of branches point of new vessels and the tube length of new vessels in comparison to cells treated with exogenous VEGF-A (Figures 7B–H).

## Discussion

Blood retinal barrier breakdown is a hallmark of diabetic retinopathy. The BRB is a tight and limitative barrier that manages the flux of ions, proteins, metabolic waste compounds, and water flow through the retina, and consists of two distinct regions, the inner BRB (iBRB) and outer BRB (oBRB). The iBRB is established by tight junctions between retinal capillary endothelial cells, surrounded by pericytes and supported by glial cells (Cunha-Vaz et al., 2011; Frey and Antonetti, 2011). The outer BRB (oBRB) is formed by retinal pigmented epithelial cells connected by tight junction proteins, which regulate transport between the choriocapillaris and the retina. Both iBRB and oBRB include tight junction proteins (TJs) (i.e., occludin, claudin family ,and zonula occludens proteins) and adherens junction proteins (i.e., VE-cadherin) (Cunha-Vaz et al., 2011; Frey and Antonetti, 2011). Hyperglycemia, oxidative stress and inflammation are detrimental events that compromise the stability and the expression of those proteins (Tien et al., 2013; Yuan et al., 2014; Platania et al., 2019). The protective effects of vitamin D_3_ have been studied in different pathological systems, including eye diseases (Jia et al., 2019; Gouni-Berthold and Berthold, 2021; Johansson et al., 2021; Plesa et al., 2021; Bakhshaee et al., 2022). Beyond the role of vitamin D_3_ in calcium and bone homeostasis, several evidence highlight the anti-inflammatory, antioxidant and anti-angiogenic activity of this natural compound (Saad El-Din et al., 2020; Ghanavatinejad et al., 2021). Recently, the attention has been focused on the correlation between vitamin D_3_ deficiency and diabetic retinopathy progression (Aksoy et al., 2000; Kaur et al., 2011; Lu et al., 2018), although the mechanism behind its effect on DR pathogenesis is not so clear. It has been hypothesized that vitamin D_3_ deficiency has a role in type 1 and type 2 diabetes pathogenesis; in particular, different studies highlighted the leading role of vitamin D receptor (VDR) in maintenance normoglycemia, and the alteration of VDR function has been linked to insulin resistance (Zeitz et al., 2003; Oh et al., 2015; Ni et al., 2016). Moreover, different allelic variations in vitamin D_3_ metabolism-related genes have been proposed as predictive markers of insulin imbalance and glucose intolerance (Ren et al., 2012; Yu et al., 2018; Shaat et al., 2020). Furthermore, vitamin D_3_ showed promising implications for diabetic retinopathy treatment, preventing inflammatory-related complications. Indeed, Lu et al. (2018) demonstrated that vitamin D_3_ inhibits the activation of inflammasome both in an *in-vitro* and *in-vivo* model of DR reducing the detrimental effects induced by high concentration of glucose. Interestingly, vitamin D_3_ also showed a relevant anti angiogenic activity in a mouse oxygen-induced ischemic retinopathy model (Albert et al., 2007). The mechanism underlying the protective effect of vitamin D_3_ in hyperglycemia-stimulated endothelial cells has not been fully elucidated. Different cytoplasmic and nuclear pathways are involved following VDR activation (Ryan et al., 2015). Incidentally, the anti-inflammatory effect of vitamin D_3_ could be related to calcium homeostasis and purinergic receptors (P2X7R) activation (Uekawa et al., 2018). On this regard, some studies demonstrated that vitamin D_3_ was able to reduce the calcium influx through P2X7R in resting human mononuclear cells and, as consequence, to down-regulate the expression of this receptor strongly linked to the exacerbation of inflammation in several diseases (Lajdova et al., 2008; Adinolfi et al., 2018). Based on this evidence the binding of vitamin D_3_ on P2X7 receptor, acting as allosteric modulator, cannot be rule out, and it is worthy of further investigations. Moreover, long-term vitamin D_3_ supplementation was shown to normalized intracellular Ca^2+^ levels in early-stage chronic kidney disease patients without any changes in intracellular calcium storage or cellular intake (Lajdova et al., 2009).

In the present study, vitamin D_3_ was able to counteract the effects mediated by inflammatory processes induced by high concentrations of glucose. In fact, we found a relevant rescue in the BRB integrity of retinal endothelial cells mediated by vitamin D_3_ in HG conditions with restored levels of junction proteins. As expected, the stimulation with HG significantly reduced TEER values after 48 h compared to control cells (Figure 2). The Na-F permeability test confirmed the BRB integrity impairment after HG treatment, thus mimicking the clinical features of DR patients (Fresta et al., 2020; Nian et al., 2021). Data reported in Figures 3, 4 indicate that vitamin D_3_ treatment reduced paracellular permeability in presence of hyperglycemia by preventing the HG-induced decrease of junction protein levels, ZO-1 and VE-cadherin, restoring their central role regarding the tight and the adherens junctions, respectively. Similarly, Won S. et al. demonstrated that vitamin D_3_ treatment was able to prevent hypoxia/reoxygenation-induced blood-brain barrier disruption through VDR-mediated NF-κB signaling pathways, in an *in vitro* model of blood brain barrier (Won et al., 2015). In our model, the protective effect of vitamin D_3_ against HG can be ascribed to its capability to block inflammatory processes that underlie the pathogenesis of diabetic retinopathy (Forrester et al., 2020). We evaluated the effects of vitamin D_3_ regarding the mRNA levels of inflammatory cytokines, ICAM-1, IL-1β, and TLR- 4 in endothelial cells treated with HG. As previously reported, HG treatment led to a significant increase in the pro-inflammatory cytokines mRNA levels, as well as TLR-4 (Xie et al., 2014; Wang et al., 2018; Zhou et al., 2019; Giurdanella et al., 2021). It has been demonstrated that high glucose promotes the activation of TLR (2/4) and, through myeloid differentiation proteins (MyD88)-dependent and -independent signaling pathway, it stimulates the release of inflammatory mediators (Devaraj et al., 2008; Dasu and Jialal, 2011), which are also significantly increased in the vitreous fluid of DR patients (Boss et al., 2017; Iyer et al., 2021; Wu et al., 2021). Our data (Figure 5) are in line with other evidence about the stimulation of TLR-4 pathway exerted by HG (Pahwa, Nallasamy and Jialal, 2016). Moreover, we have previously demonstrated that HG mediate cell damage through the activation of MAPK/NFκB axis through the phosphorylation of both these proteins (Giurdanella et al., 2017; Giurdanella et al., 2020; Lazzara et al., 2019). On these bases, here we tested the HG-induced cytokines mRNA up-regulation, as direct consequence of the activation of ERK/NFκB pathway; in our model, vitamin D_3_ clearly reduced the phosphorylation of ERK protein and counteracted the nuclear translocation of phosphorylated p65 NFκB subunit and the cognate increase in cytokine mRNA levels (Figures 5, 6). Vitamin D_3_ could exert a pleiotropic anti-inflammatory activity considering its capability to counteract different pathways; this point would certainly need further investigation. We cannot rule out the hypothesis that vitamin D_3_ could interfere with the activation of ROS-related HMGB1-TLR4 signaling, described to induce endothelial dysfunction in presence of HG (Rao et al., 2017; Zhang et al., 2018; Fernandez-Robredo et al., 2020; Huang et al., 2020). Moreover, our results could be consistent with a putative contribution of vitamin D_3_ in calcium homeostasis through the involvement of the purinergic system (P2X7R) that we found involved in high glucose-induced retinal endothelial damage (Platania et al., 2017). Our *in-vitro* findings confirm the effect of vitamin D_3_ as inhibitor of retinal neo-angiogenesis. It has well demonstrated, that vitamin D_3_ hampered VEGF-induced endothelial cell sprouting and elongation (Mantell et al., 2000; Albert et al., 2007; Jamali et al., 2019). It has also been shown that vitamin D_3_ treatment inhibited VEGF-induced activation of VEGFR-2, ERK and Akt pathway (Kim et al., 2017). Indeed, in our study we found that vitamin D_3_ affects the pro-angiogenic activity of VEGF-A on HRECs, and significantly reduced VEGF-A mRNA levels elicited by high levels of glucose (Figure 7).

In conclusion, we provided new evidence on the role of vitamin D_3_ in an *in vitro* model of DR using human retinal endothelial cells. The BRB integrity, significantly compromised by high glucose exposure, was restored by vitamin D_3_ treatment. These data suggest that vitamin D_3_ could be a good candidate to counteract inflammation in several retinal conditions and warranting further clinical evaluation of the efficacy profile.

## Data Availability

The original contributions presented in the study are included in the article/supplementary materials, further inquiries can be directed to the corresponding author.
